# Novel Circoviruses from Birds Share Common Evolutionary Roots with Fish Origin Circoviruses

**DOI:** 10.3390/life12030368

**Published:** 2022-03-03

**Authors:** Enikő Fehér, Eszter Kaszab, Krisztina Bali, Márton Hoitsy, Endre Sós, Krisztián Bányai

**Affiliations:** 1 Veterinary Medical Research Institute, H-1143 Budapest, Hungary; kaszab.eszter@vmri.hu (E.K.); bali.krisztina@vmri.hu (K.B.); banyai.krisztian@vmri.hu (K.B.); 2 Conservation and Veterinary Services, Budapest Zoo and Botanical Garden, H-1164 Budapest, Hungary; drhoitsy@zoobudapest.com (M.H.); drsos.endre@zoobudapest.com (E.S.); 3 Department of Pharmacology and Toxicology, University of Veterinary Medicine, H-1078 Budapest, Hungary

**Keywords:** circovirus, wild birds, genome sequencing, next generation sequencing, novel species

## Abstract

Circoviruses occur in a variety of animal species and are common pathogens of mammalian and avian hosts. In our study internal organ samples of wild birds were processed for screening of circoviral sequences. Two novel viruses were identified and characterized in specimens of a little bittern and a European bee-eater that suffered from wing injuries, were weakened, had liver or kidney failures, and finally succumbed at a rescue station. The 1935 nt and 1960 nt long viral DNA genomes exhibited a genomic structure typical for circoviruses and were predicted to encode replication-associated protein in the viral strand, and a capsid protein in the complementary strand of the replicative intermediate DNA form. The genome of the newly described viruses showed 37.6% pairwise identity with each other and ≤41.5% identity with circovirus sequences, and shared a common branch with fish, human and Weddel seal circoviruses in the phylogenetic tree, implying evolutionary relationship among the ancestors of these viruses. Based on the results the little bittern and European bee-eater circoviruses represent two distinct species of the *Circovirus* genus, *Circoviridae* family.

## 1. Introduction

The increasing number of recently discovered viruses with circular replication-associated protein (Rep)-encoding single stranded (CRESS) DNA genomes has highlighted the diversity and helped to improve the classification of this group of viruses in the past decade. Members of the *Bacilladnaviridae*, *Geminiviridae*, *Nanoviridae, Genomoviridae*, *Redondoviridae*, *Smacoviridae* and *Circoviridae* families, as well as a number of unclassified viruses are referred as eukaryotic CRESS DNA viruses and are associated with plants, diatoms, fungi and animals [1,2]. The Rep of the eukaryotic CRESS DNA viruses encode an endonuclease and a superfamily 3 helicase domain that may initiate rolling circle replication (RCR) of these viruses [1,2].

Circoviruses are genetically well characterized animal CRESS DNA viruses taxonomically belonging to the *Circovirus* genus within the *Circoviridae* family, *Cirlivirales* order, *Cressdnaviricota* phylum [1,2]. The ambisense genomes of circoviruses are ~1600–2200 nt in length and code for the Rep in the viral strand and capsid (Cp) protein in the complementary DNA strand produced during the RCR [2]. Circoviral nucleic acid has been detected in internal organs and feces of mammals, birds, fishes, and in insects [2]. Some mammalian and avian circoviruses, such as porcine circovirus 2 and 3, beak and feather disease virus, pigeon circovirus, goose circovirus, duck circovirus, finch circovirus, and canary circovirus may induce fatal diseases in the respective host, while others, for example porcine circovirus 1, are considered as non-pathogenic agents [3,4,5,6,7,8,9,10,11,12]. Due to the limited data the exact host (e.g., for feces-associated viruses) and pathogenic role has not been revealed for many viruses in this genus [3,4,5,6,7,8,9,10,11,12].

In general, circovirus infection of birds may lead to lethargy, weight-loss, feathering disorders, beak deformities, diarrhea and internal organ failures [3,4,6,7,8,9,10,11,12]. The viruses may generate immunosuppression via lymphocyte depletion, thus the vertebrate host may become susceptible for severe secondary infections [4,5,6,7,9,10]. In addition to the above mentioned, circoviruses of unknown etiology have been also described in birds, including canary circovirus, gull circovirus, penguin circovirus, raven circovirus, starling circovirus, swan circovirus and zebra finch circovirus [11].

Weakened and diseased animals may often get trauma or may become prey of predators. In this study, samples of injured and later died wild birds with poor body condition and traumatic lesions were processed for seeking viruses of the *Circoviridae* family. Following complete genome analysis two of the three identified CRESS DNA viruses were found to be representatives of novel circovirus species.

## 2. Materials and Methods

### 2.1. Sample Preparation

All samples originated from wild birds weakened of unknown reasons and/or suffering from traumatic injuries. The birds were rescued and transported to the Zoo and Botanical Garden, Budapest, Hungary, 2019, where they received medical treatment. Small fractions of organ samples (liver, kidney, spleen, bursa of Fabricius, thymus) were taken from the succumbed birds (*n* = 32, Table 1) and then homogenized in 1 mL phosphate buffered solution using TissueLyzer LT (Qiagen, Hilden, Germany) device. Then, samples were centrifuged at 10.000× *g* for 5 min. The homogenates were mixed for each bird and the nucleic acid was extracted with ZiXpress-32^®^ Viral Nucleic Acid Extraction Kit and ZiXpress-32^®^ Automated Nucleic Acid Purification Instrument (Zinexts Life Science Corp., New Taipei City, Taiwan).

### 2.2. Circovirus Specific PCR and Complete Genome Amplification

The nucleic acid samples were tested for viral DNA of the *Circoviridae* family with nested PCR [13,14,15,16]. Twenty μL PCR mixtures contained 250 μM dNTP mix, 250 nM primers, 1× DreamTaq Green buffer, 0.5 U DreamTaq DNA Polymerase (Thermo Fisher Scientific, Waltham, MA, USA) and 1 μL of the extracted nucleic acid. The cycling protocols consisted of a denaturation step at 95 °C for 3 min, 40 cycles of denaturation at 95 °C for 30 s, annealing at 52 °C (first round of nested PCR, CV-F1 5′-GGIAYICCICAYYTICARGG-3′ and CV-R1 5′-AWCCAICCRTARAARTCRTC-3′ primers) and 56 °C (second round of nested PCR, CV-F2 5′-GGIAYICCICAYYTICARGGITT-3′ and CV-R2 5′-TGYTGYTCRTAICCRTCCCACCA-3′ primers) for 30 s and extension at 72 °C for 1 min, followed by a final extension step at 72 °C for 10 min [13,14,15,16]. PCR products of ~400 bp in length were purified from agarose gel with Geneaid Gel/PCR DNA Fragments Extraction Kit (Geneaid Biotech, Taipei, Taiwan) and were directly sequenced with the CV-F2 and CV-R2 primers used in the second round of nested PCR.

Based on the sequences obtained by Sanger method, back-to-back PCR primers (CV_20190722-2_F 5′-CACGTAACTGGAAGACGGAAGTAC-3′ and CV_20190722-2_R 5′-CTTGCACAAGTCCAGACATGTTC-3′ for the little bittern sample; CV_20190809-1_F 5′-ATCGAGTCTGCTGTAGAGATCCTTCG-3′ and CV_20190809-1_R 5′-ATCCGTGCGT TTCCCTTGAGAG-3′ for the European bee-eater sample) were designed and utilized for complete genome amplification [13,14,15]. Twenty-five μL PCR mixture contained 1× Phusion Green HF buffer, 200 μM dNTP mix, 200 nM primers and 0.25 U Phusion DNA Polymerase (Thermo Fisher Scientific) as well as 1 μL of the extracted nucleic acid. The cycling protocols consisted of denaturation step at 98 °C for 30 s, 45 cycles of denaturation at 98 °C for 10 s, annealing at 61 °C for 30 s and extension 72 °C for 1 min, followed by a final extension step at 72 °C for 10 min. The PCR products were purified from agarose gel with Geneaid Gel/PCR DNA Fragments Extraction Kit (Geneaid Biotech, Taipei, Taiwan) and were submitted for next generation sequencing.

### 2.3. Next-Generation Sequencing

DNA libraries were prepared for next generation sequencing using Illumina^®^ Nextera XT DNA Library Preparation Kit (Illumina, San Diego, CA, USA) and Nextera XT Index Kit v2 Set A (Illumina). The amplified virus genomic DNA samples were diluted to 0.2 ng/μL in nuclease-free water in a final volume of 2.5 μL. Five microliters of Tagment DNA buffer and 2.5 μL of Amplicon Tagment Mix were used during tagmentation step. The samples were incubated at 55 °C for 6 min in a GeneAmp PCR System 9700 (Thermo Fisher Scientific). Neutralization was performed for 5 min at room temperature after pipetting of 2.5 μL of Neutralize Tagment buffer to the mixture. The i5 and i7 index primers were incorporated into the library DNA via PCR (cycling protocol: 72 °C for 3 min, 95 °C for 3 min, 12 cycles of the steps 95 °C for 10 s, 55 °C for 30 s, and 72 °C for 30 s, followed by a final incubation at 72 °C for 5 min). The PCR mixture contained 7.5 μL of the Nextera PCR Master Mix, 2.5 μL each of the primers and the tagmented DNA samples. The libraries were purified with Geneaid Gel/PCR DNA Fragments Extraction Kit (Geneaid Biotech) and were pooled to a final concentration of 1.5 pM. The library pool was sequenced using NextSeq 500/550 Mid Output flow cell and an Illumina^®^ NextSeq 500 sequencer platform (Illumina).

### 2.4. Software

The Geneious Prime v 2020.2.4 (Biomatters Ltd., Auckland, New Zealand) was applied for de novo assembly of the sequence reads. The sequences were edited and aligned with MUSCLE option of the AliView and Geneious Prime software [17]. The estimation of recombination was carried out with six methods (RDP, GENECONV, BootScan, MaxChi, Chimaera, SiScan) of the RDP5 software, involving reference sequences of all circovirus species and the novel sequences [18]. Phylogenetic analyses were performed with the same sequence data set using the PhyML software, the GTR + G + I model and SH-like branch support [19]. The phylogenetic trees were visualized and edited with the MEGA6 software [20]. Pairwise nt and aa identities were calculated and represented with Geneious Prime and SDT v 1.2 software [21].

## 3. Results and Discussion

Circovirus-like *rep* sequences were amplified with nested PCR in 3 of the 32 internal organ specimens (9.4%) (Table 1). One of these originated from liver/kidney/bursa of Fabricius mixture of a Eurasian sparrowhawk (*Accipiter nisus*) transported to the rescue station with trauma. The sequence of the PCR product shared high nt identity (96.5%) with the *rep* of starling circovirus as revealed by BLAST analysis. Additional *rep* sequences were detected in the kidney/liver/bursa of Fabricius of a little bittern (*Ixobrychus minutus*) and in the kidney/liver of a European bee-eater (*Merops apiaster*). These birds also had traumatic injuries and succumbed a few days after their admission to the hospital. The *rep* sequences amplified from the little bittern and European bee-eater showed ≤74.9% (≤38% query cover) and ≤66.26% nt identity (≤77% query cover), respectively, with circoviral sequences obtained in previous studies by metagenomics analysis of bird cloacal swab samples and with representative strains of established avian circovirus species.

**Figure 1:**
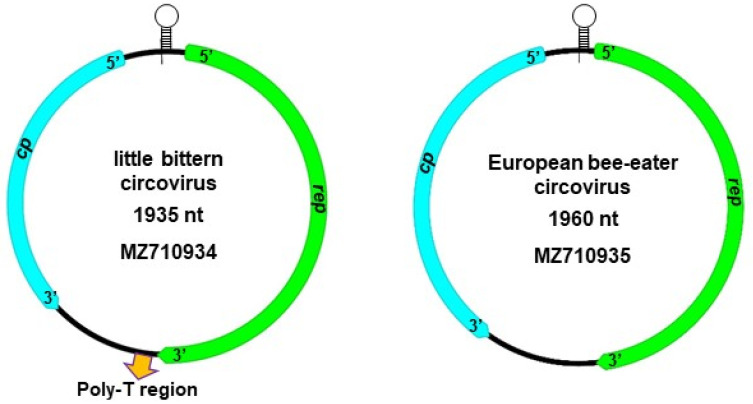
Schematic illustration of genomic structure of the little bittern circovirus and European bee-eater circovirus. The stem-loops represent the TAGTATTAC nonanucleotide motif and the flanking inverted repeats at the replication origo.

The complete genome sequence of the two putative novel circoviruses was determined with whole genome amplification and next generation sequencing. Altogether, 740,218 (at mean sequencing depth of 31,040×) and 927,115 (at mean sequencing depth of 39,918×) reads mapped to the homologous de novo assembled little bittern and European bee-eater origin circoviral sequences, respectively. The length of the genome was 1935 nt for the little bittern and 1960 nt for the European bee-eater origin CRESS DNA virus. The structure of the genomes corresponded to that of circoviruses, thus the viruses were named little bittern circovirus (BitternCV) and European bee-eater circovirus (Bee-eaterCV) (Figure 1 and Table 2) [2,22].

Both genomes contained two main open reading frames (ORFs). Using TAGTATTAC nonanucleotide motif of the putative replication origo for gene localization, 948 nt (315 aa) and 912 nt (303 aa) long Rep coding genes were identified in the viral DNA strand, and 630 nt (209 aa) and 723 nt (240 aa) long capsid protein coding (Cp) genes were predicted to be encoded on complementary replicative DNA strand of the BitternCV and Bee-eaterCV, respectively (Figure 1) [2,22]. The 5′ intergenic region (IR; 128 nt long for the BitternCV and 100 nt long for the Bee-eaterCV), located between the 5′ ends of the *rep* and *cp*, encoded the nonanucleotide motif. The encompassing inverse repeats (12 nt long for BitternCV and 13 nt long for Bee-eaterCV) suggested loop formation. The 229 nt long 3′ IR of the BitternCV contained a poly-T region of 27 nt that was previously described solely in the IR of bat-associated circovirus 10 and 13 genomes (Figure 1). Poly(T) sequences could not be found in the 225 nt long 3′ IR of the Bee-eaterCV. The exact function of the poly-T tract as part of the circoviral genome is unknown, but it is conceivable that this motif may have a role in (post-)transcriptional processes [23,24]. Investigation of the 3′ end of the circoviral *rep* and *cp* suggested the presence of polyadenylation signals in the BitternCV, Bee-eaterCV, and previously described circoviral genomes as well, that was often AAUAAA for the *rep* but highly varied for the *cp* [25,26].

The Rep of the BitternCV and Bee-eaterCV contained conserved motifs controlling the RCR processes of eukaryotic CRESS DNA viruses, including the probable N-terminal RCR (I–III) and C-terminal superfamily 3 helicase (Walker-A Walker-B, and C) motifs, as well as an arginine finger (Table 2). These motifs showed the highest similarity with that of typical for circo- and cycloviruses and imply similar course of replication for all these viruses [2,22,27,28].

Although Cp proteins of circoviruses are highly diverse, the accumulation of basic amino acids in the N-terminal region is a common feature that may be important in nuclear localization and viral DNA binding, thus in packaging into the viral capsid [2,29]. Accumulation of arginine and lysine was also characteristic to the Cp of the BitternCV and Bee-eaterCV. Interestingly, the *cp* of the Bee-eaterCV was predicted to start with the alternative start codon TTG; the usage of start codon other than ATG could be often identified for the *cp* of circoviruses submitted to the GenBank, including avian circoviruses (beak and feather disease virus, finch, gull, penguin, pigeon, raven, and zebra finch circovirus), avian-like circovirus (*Tick associated circovirus 1*), tick circovirus (*Tick associated circovirus 2*) barbel circovirus, Culex circovirus-like virus (*Mosquito associated circovirus 1*), chimpanzee faeces associated circovirus (*Chimpanzee associated circovirus 1*), rodent circoviruses and bat associated circoviruses.

Preceding phylogenetic analysis, evaluation of potential interspecies recombination was performed using complete genome sequences representing different circoviral species, but none of the novel avian circoviruses were involved into any predicted events. Statistical support of recombination (*p*-value ranging between 10^−6^ and 10^−14^ for all of the six applied methods, 10^−13^–10^−14^ for three methods) was detected only for variable rodent circoviruses, that affected a ~180 nt long part of these genomes covering the 5′ end of the *rep* and downstream the 5′ IR. In this case the results suggested that rodent associated circovirus 3 may be the recombinant descendant of the rodent associated circovirus 1 and 4, or an ancestor of these viruses. Intraspecies recombination may also affect evolution of circoviruses, including beak and feather disease virus, porcine circovirus 2, and canine circovirus [30,31,32,33]. However, these processes cannot be represented with this sequence collection.

**Figure 2:**
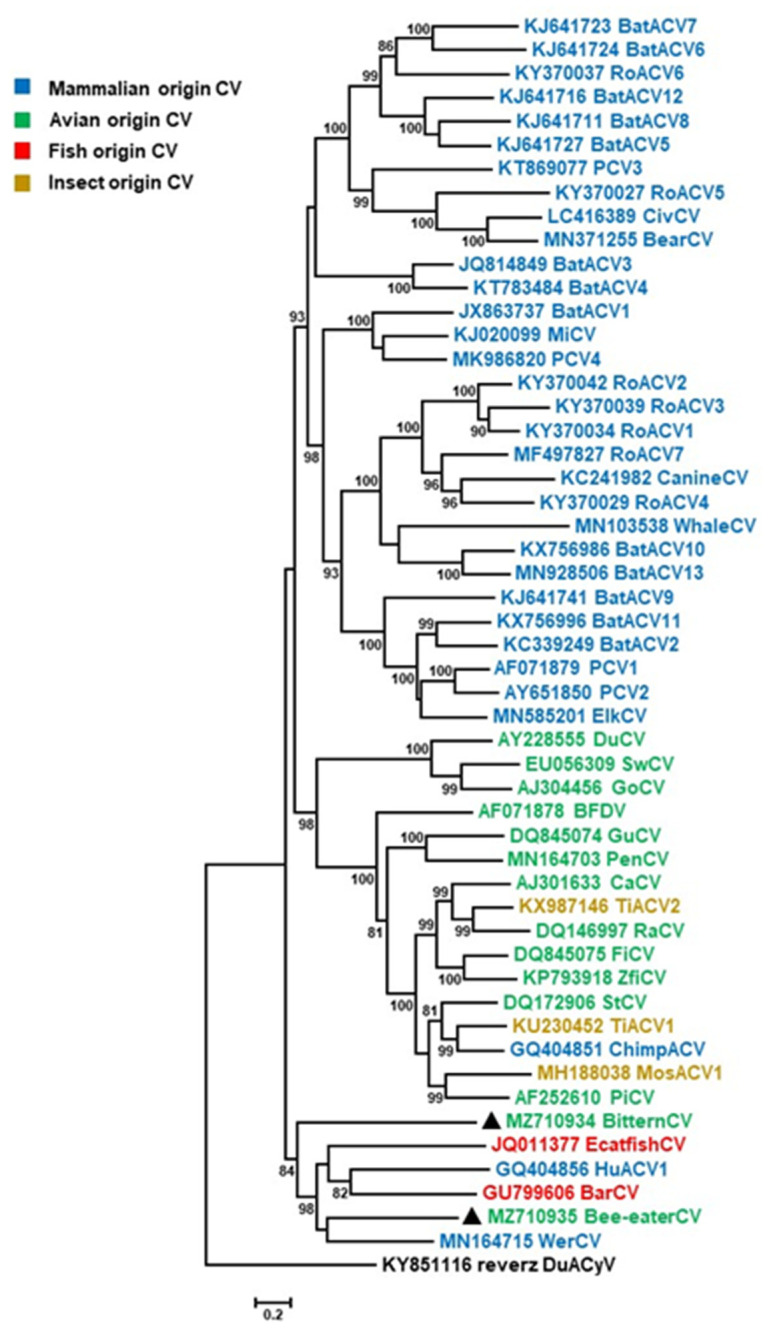
Maximum likelihood phylogenetic tree of representative complete genome sequences of circoviruses using the PhyML software, and applying GTR + G + I model and aLRT SH-like branch support. Branch support values lower than 80 were hidden. Reverse complement of a cyclovirus genomic sequence (duck associated cyclovirus 1, GenBank accession no. KY851116) was used as root for the tree. The novel circoviruses, the little bittern circovirus and European bee-eater circovirus, are highlighted with black triangles.

In addition to the genomic organization, phylogenetic analyses confirmed that the novel CRESS DNA viruses belong to circoviruses. Both the BitternCV and Bee-eaterCV clustered together with barbel circovirus (BarCV), European catfish circovirus (EcatfishCV), Weddel seal Ross Sea associated circovirus (WerCV) and human faeces associated circovirus (HuACV1) (Figure 2). Sequences of this branch were connected with deeper nodes that suggested an ancient evolutionary relationship among these six circoviral genomes. Both fish circoviruses originated from Hungary; BarCV was described in barbel fry (*Barbus barbus*) hatched in a fish farm, while EcatfishCV genome was amplified from organ specimens of European catfish (*Silurus glanis*) carcasses collected in Lake Balaton [34,35]. These papers did not report the presence of agents other than circovirus in these fish. The HuACV1 and WerCV strains were detected in human stool sample in Tunisia and in the feces of a Weddel seal *(Leptonychotes weddellii*) in the Antarctica, respectively [16,36]. Although the novel avian origin circovirus strains and the closest references are only distantly related, it cannot be ruled out that the aquatic environment might be a source of possible common ancestor(s). However, identification of the exact host(s) is still to be clarified.

**Figure 3:**
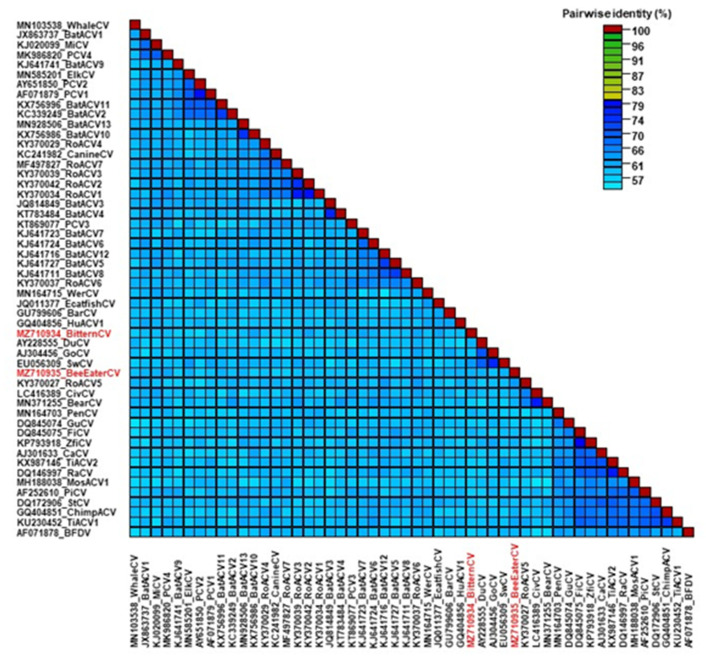
Complete genome sequence based pairwise identity matrix of representative circovirus sequences using SDT v1.2 software. The novel circoviruses, the little bittern circovirus and European bee-eater circovirus, are highlighted with red in the species list.

Based on calculations of pairwise identity values BarCV, EcatfishCV, WerCV, HuACV1 and avian circoviruses were the closest relatives of the BitternCV and Bee-eaterCV, sharing a maximum of 41.5% genome-wide nt identities with the avian origin viruses. The BitternCV and Bee-eaterCV were also only distantly related to each other with 37.6% nt identity for the complete genome (Figure 3). The *rep* of the two novel avian circovirus sequences showed 51.6% nt and 48.2% aa identity with each other, and ≤57.7% nt and ≤57.8% aa identities with the reference sequences. The *cp* of the BitternCV and Bee-eaterCV shared 34.3% nt and 17.6% aa identity with each other and ≤39.7% nt and ≤32.0% aa identities with the selected references.

At present, 49 virus species belong to the *Circovirus* genus. The demarcation criteria set by the International Committee on Taxonomy of Viruses for circovirus species include the appropriate genome structure and a maximum genome-wide pairwise nt identity value of 80% [2]. Our results confirmed that BitternCV and Bee-eaterCV belong to two distinct novel circovirus species, tentatively named *Little bittern circovirus* and *European bee-eater circovirus*. Both viruses were detected in internal organ samples, thus the aforementioned avian species may be susceptible hosts for the replication of their respective viruses. Nonetheless, other experiments are needed to prove this association. The quality of the samples collected for this study were considered not ideal for individual analysis of the organs and for histopathological examinations, thus the site of infection could not be defined. Another drawback of this and other circovirus-related studies is the lack of suitable homologous cell cultures [37,38,39]. Resolving propagation could greatly advance research of biological properties of potentially pathogenic circoviruses.

The little bittern and European bee-eater, tested positive for circoviruses, were transported to the rescue station and died presumably by traumatic injuries. However, upon gross pathology weight loss and enlarged liver with lesions, and degenerated kidneys were also recorded for the birds, respectively. These findings match with the signs generally connected to avian circoviruses, i.e., weakness, lethargy, growth retardation, lymphoid cell depletion and internal organ failures [3,4,6,7,8,9,10,11,12]. Thus, although more precise pathological and histological examinations or testing of other pathogens were not performed, the etiological role of the circoviruses in these disorders could not be ruled out. Nevertheless, it cannot be decided whether the traumatic injury was the consequence of the infection and the resulting weakness, or vice versa. As avian circoviruses have been characterized as immunosuppressive agents and may have direct pathogenic role in birds [4,6,7,9,10], further investigation about host spectrum and pathogenicity of the little bittern and European bee-eater circoviruses would be of great interest.

## Figures and Tables

**Table 1:** The host and type of specimens used in this study. Samples with detected circoviral sequence are shown in bold. B: bursa of Fabricius; K: kidney; L: liver; S: spleen; T: thymus. NA, data not available.

Bird Species	Sample	Data about the Rescued Bird
Common blackbird, *Turdus merula*	B,K,L,T	Leg injury
	K,L	Traumatic injuries, internal bleeding
	K,L,S	Hemorrhagic fluid in the thoracoabdominal cavity
Common buzzard, *Buteo buteo*	K,L,S	Caseonecrotic granulomas in lung, liver, and gizzard; mycobacteriosis
	K,L	Electric shock, necrotizing leg
Common crane, *Grus grus*	Lesion	Signs of pox virus infection
Common house martin, *Delichon urbicum*	K,L	NA
	L	Eye lesions, small, pale kidneys
Common kestrel, *Falco tinnunculus*	K,L	Traumatic injuries
	K,L	Wing injury
	B,K,L	NA
	K,L	Electric shock
	K,L	Electric shock
Common kingfisher, *Alcedo atthis*	K,L	NA
Common pheasant, *Phasianus colchicus*	K,L,S	NA
Eurasian woodcock, *Scolopax rusticola*	K,L	Shot injury of the breast
**European bee-eater, *Merops apiaster***	K,L	**Wing injury, weight loss, degenerated kidneys**
European Green Woodpecker, *Picus viridis*	B,K,L,S	Head injury
European honey buzzard, *Pernis apivorus*	K,L	Traumatic injuries
**Eurasian sparrowhawk, *Accipiter nisus***	B,K,L	**Wing injury**
	K,L	Weight loss, bleeding in the stomach
Great cormorant, *Phalacrocorax carbo*	K,L	Traumatic injuries, tested positive for polyomavirus
Great spotted woodpecker, *Dendrocopos major*	K,L	Traumatic injuries
Grey heron, *Ardea cinerea*	K,L	Necrotizing wing, visceral gout
**Little bittern, *Ixobrychus minutus***	B,K,L	**Wing injury, poor body condition, weight loss, enlarged liver with lesions**
	B,K,L	Traumatic injuries of the left body site, kidney injury
	B,K,L	Poor body condition, broken lower mandible, visceral gout
Little owl, *Athene noctua*	K,L	Poor body condition and weight loss
Mute swan, *Cygnus olor*	K,L	Weight loss, diarrhea
Tawny owl, *Strix aluco*	K	NA
	K,L	Enlarged liver, liver failure, pale kidneys
Water rail, *Rallus aquaticus*	K,L	Pale kidneys

**Table 2:** Localization of main coding and non-coding regions and sequences of conserved motifs in the little bittern and European bee-eater circovirus genomes. *rep*: replication-associated protein coding gene; *cp*: capsid protein coding gene.

	Little Bittern Circovirus	European Bee-Eater Circovirus
Genome	1935 nt	1960 nt
5′ intergenic region	nt 1859–51	nt 1894–33
Nonanucleotide	TAGTATTAC	TAGTATTAC
Stem-loop inverted repeat	CACAGGCGCCGG	GCCGAGGTGGCCG
*rep*	nt 52–999 (315 aa)	nt 34–945 (303 aa)
RCR motif I	MTLNN	FTLNN
RCR motif II	PHLQG	PHLQG
RCR motif III	YCSK	YCSK
Walker-A motif	GPPGCGKT	GPPGCGKS
Walker-B motif	VIDDF	IVDDF
Motif C	ITSN	ITSN
*cp*	nt 1858–1229 (209 aa)	nt 1893–1171 (240 aa)
3′ intergenic region	nt 1000–1228	nt 946–1170

## Data Availability

The sequence data are available in the GenBank with accession number MZ710934-MZ710935.
